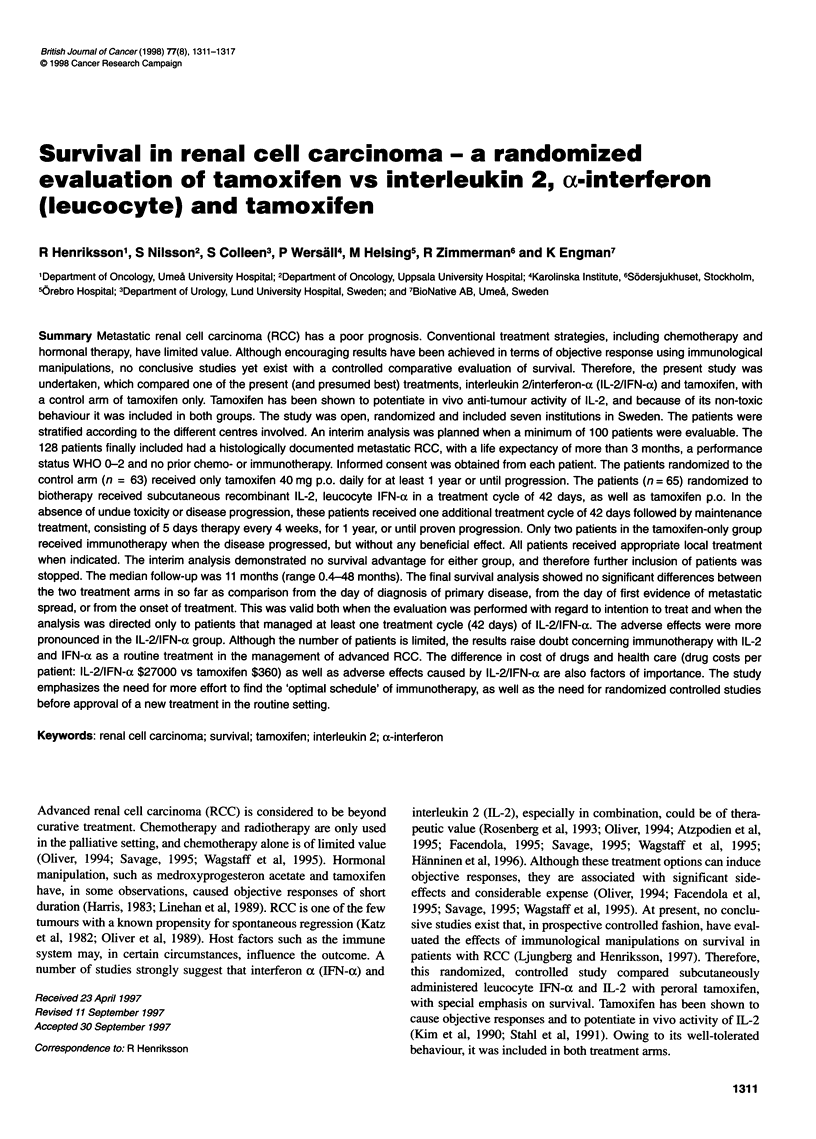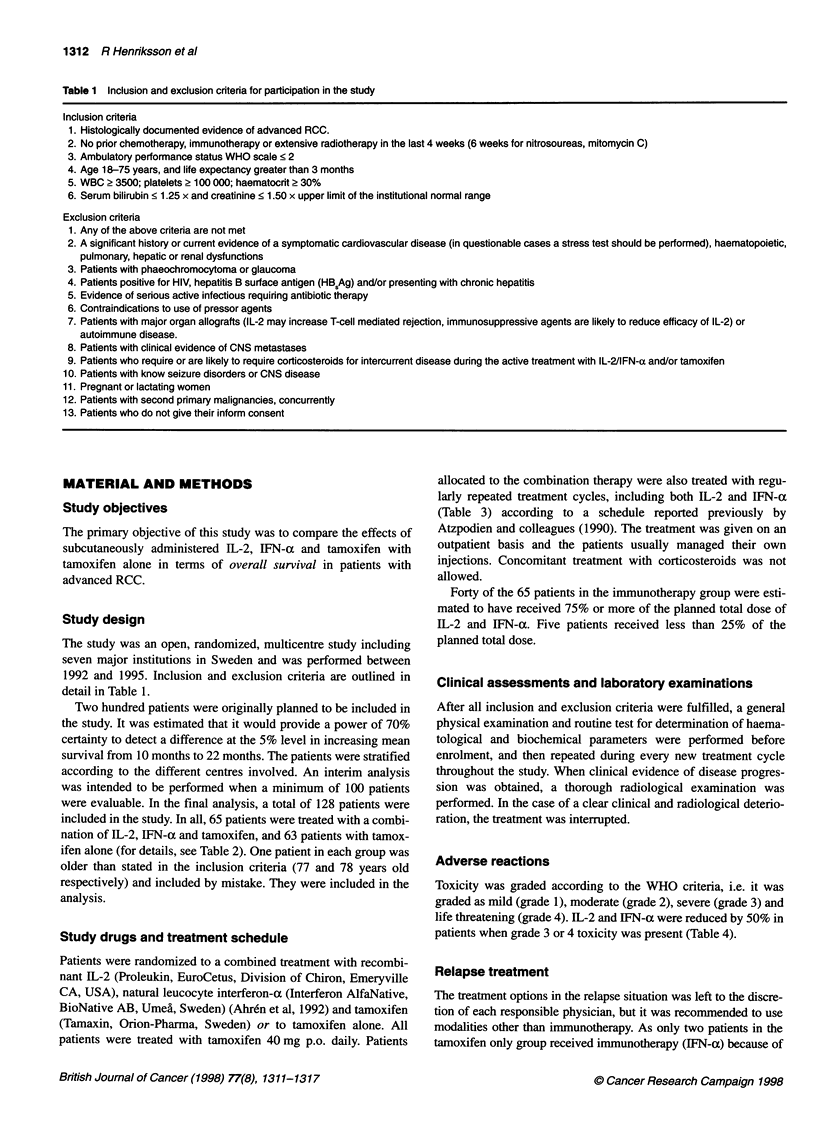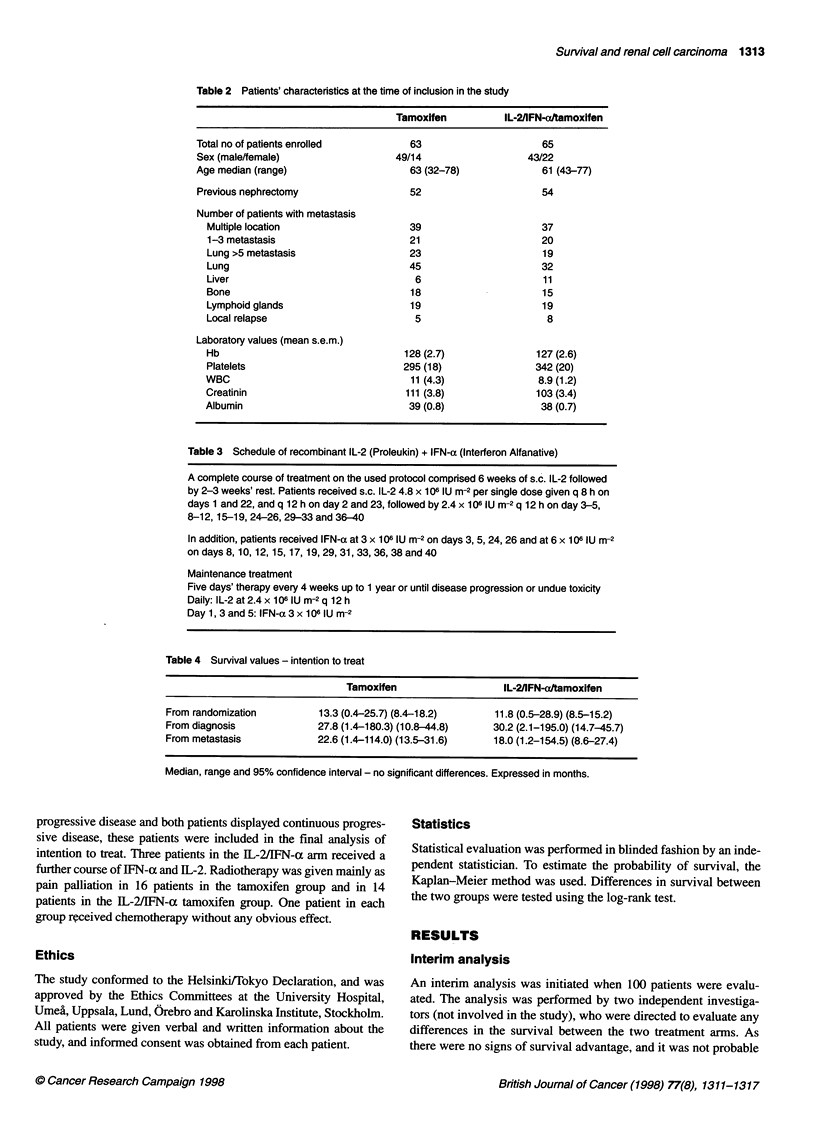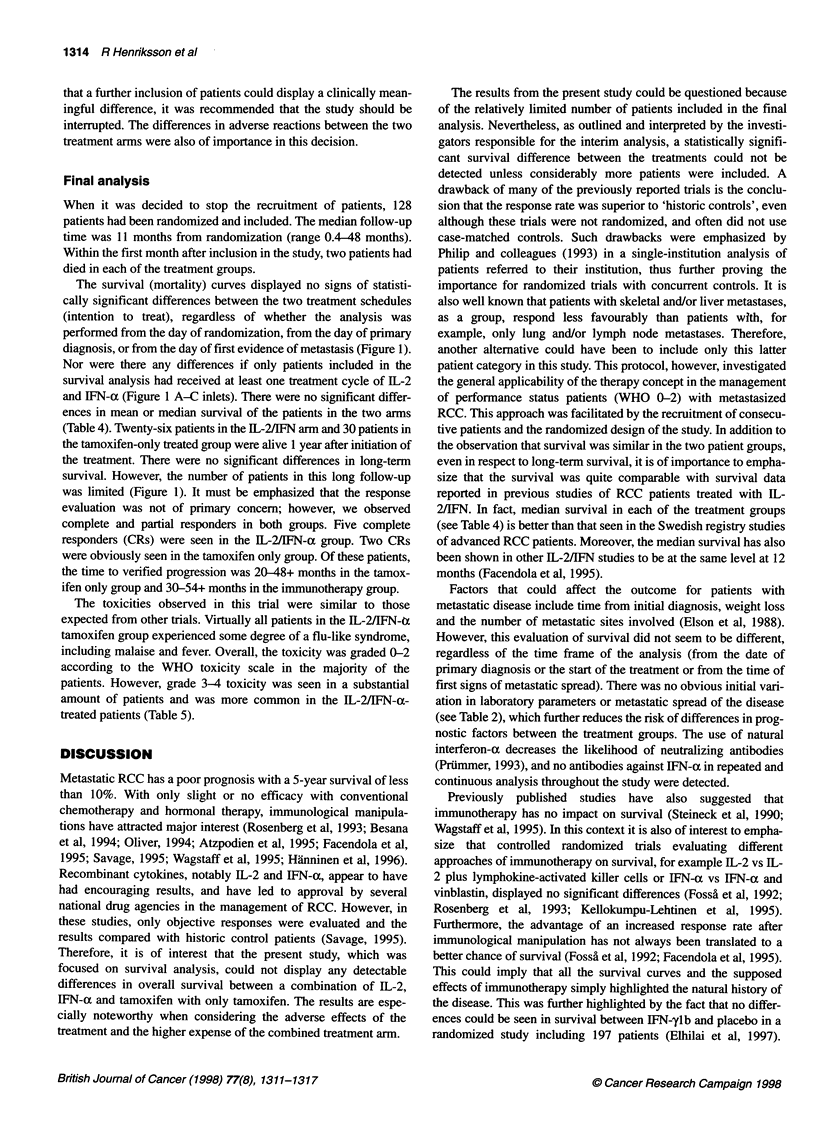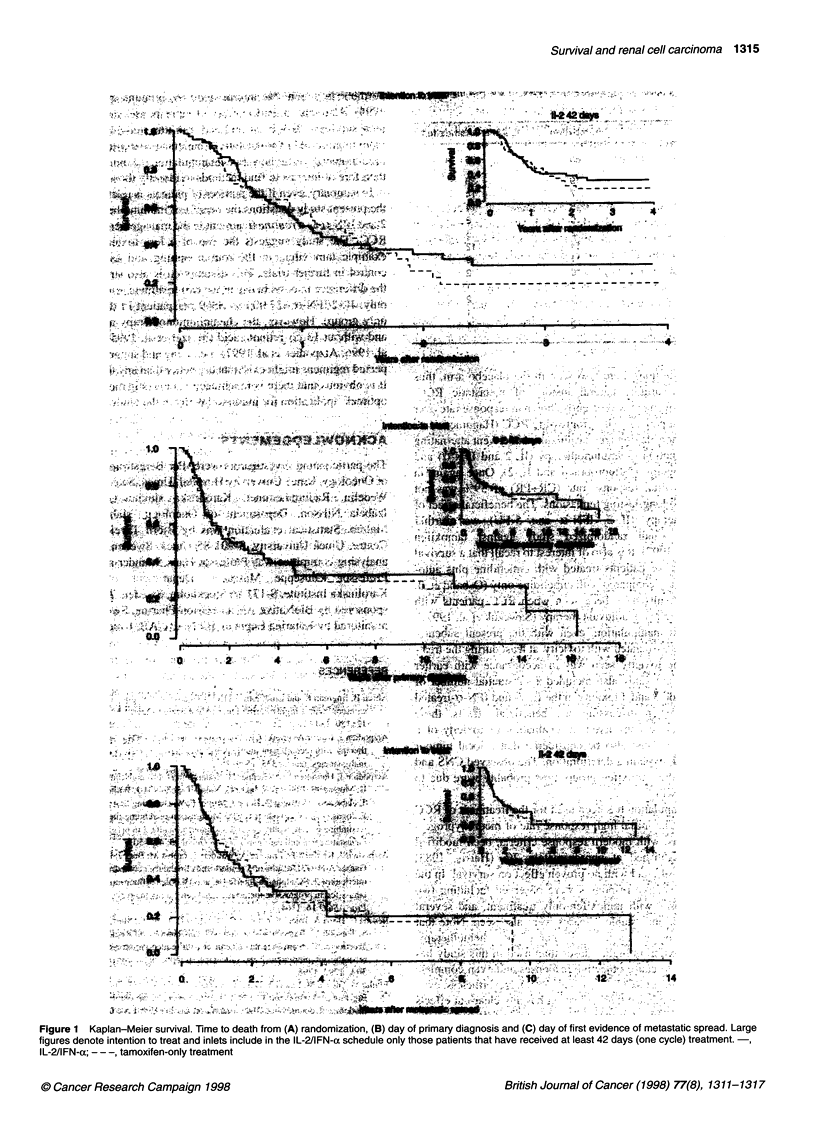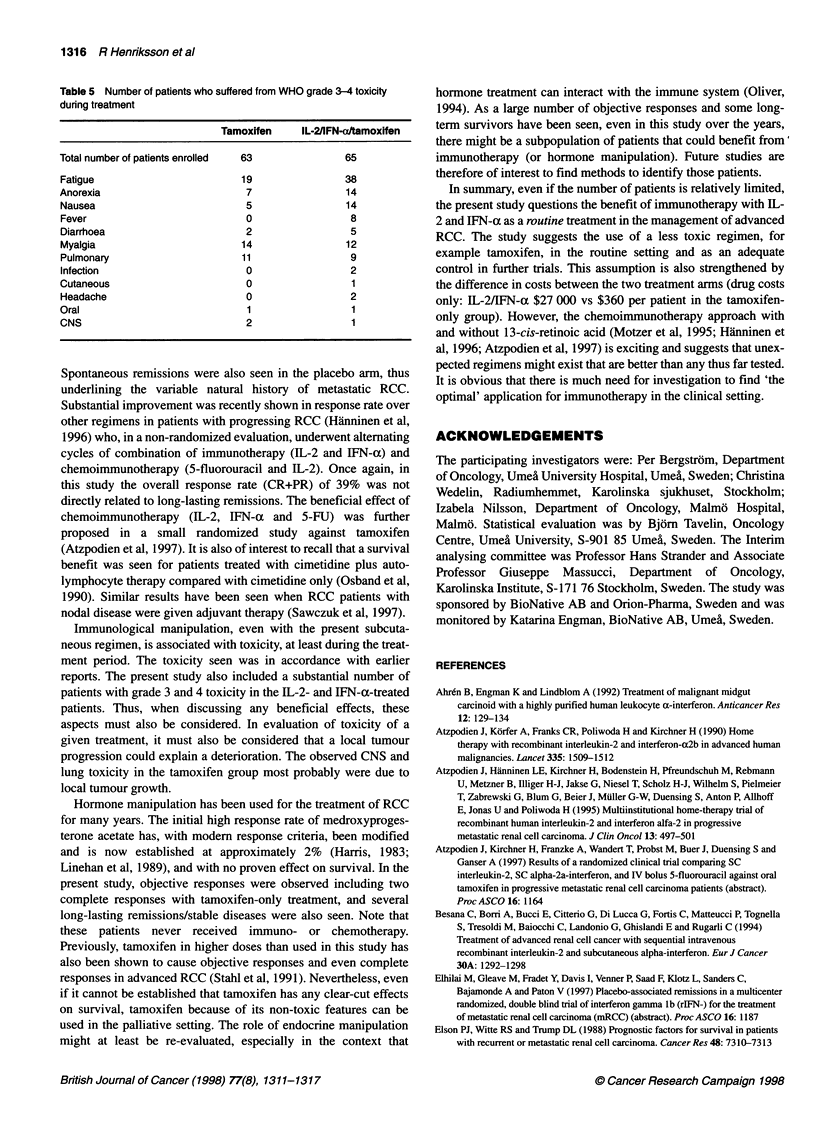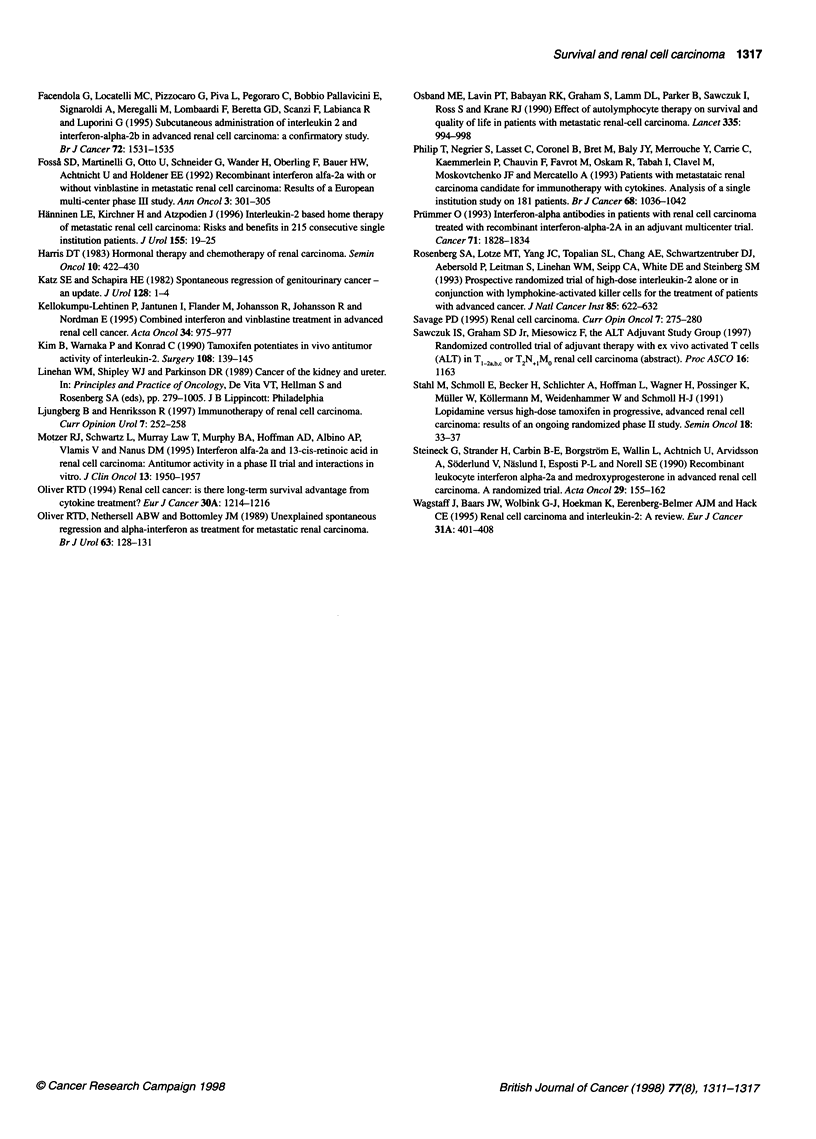# Survival in renal cell carcinoma-a randomized evaluation of tamoxifen vs interleukin 2, alpha-interferon (leucocyte) and tamoxifen.

**DOI:** 10.1038/bjc.1998.218

**Published:** 1998-04

**Authors:** R. Henriksson, S. Nilsson, S. Colleen, P. WersÃ¤ll, M. Helsing, R. Zimmerman, K. Engman

**Affiliations:** Department of Oncology, UmeÃ¥ University Hospital, Sweden.

## Abstract

Metastatic renal cell carcinoma (RCC) has a poor prognosis. Conventional treatment strategies, including chemotherapy and hormonal therapy, have limited value. Although encouraging results have been achieved in terms of objective response using immunological manipulations, no conclusive studies yet exist with a controlled comparative evaluation of survival. Therefore, the present study was undertaken, which compared one of the present (and presumed best) treatments, interleukin 2/interferon-alpha (IL-2/IFN-alpha) and tamoxifen, with a control arm of tamoxifen only. Tamoxifen has been shown to potentiate in vivo anti-tumour activity of IL-2, and because of its non-toxic behaviour it was included in both groups. The study was open, randomized and included seven institutions in Sweden. The patients were stratified according to the different centres involved. An interim analysis was planned when a minimum of 100 patients were evaluable. The 128 patients finally included had a histologically documented metastatic RCC, with a life expectancy of more than 3 months, a performance status WHO 0-2 and no prior chemo- or immunotherapy. Informed consent was obtained from each patient. The patients randomized to the control arm (n = 63) received only tamoxifen 40 mg p.o. daily for at least 1 year or until progression. The patients (n = 65) randomized to biotherapy received subcutaneous recombinant IL-2, leucocyte IFN-alpha in a treatment cycle of 42 days, as well as tamoxifen p.o. In the absence of undue toxicity or disease progression, these patients received one additional treatment cycle of 42 days followed by maintenance treatment, consisting of 5 days therapy every 4 weeks, for 1 year, or until proven progression. Only two patients in the tamoxifen-only group received immunotherapy when the disease progressed, but without any beneficial effect. All patients received appropriate local treatment when indicated. The interim analysis demonstrated no survival advantage for either group, and therefore further inclusion of patients was stopped. The median follow-up was 11 months (range 0.4-48 months). The final survival analysis showed no significant differences between the two treatment arms in so far as comparison from the day of diagnosis of primary disease, from the day of first evidence of metastatic spread, or from the onset of treatment. This was valid both when the evaluation was performed with regard to intention to treat and when the analysis was directed only to patients that managed at least one treatment cycle (42 days) of IL-2/IFN-alpha. The adverse effects were more pronounced in the IL-2/IFN-alpha group. Although the number of patients is limited, the results raise doubt concerning immunotherapy with IL-2 and IFN-alpha as a routine treatment in the management of advanced RCC. The difference in cost of drugs and health care (drug costs per patient: IL-2/IFN-alpha $27000 vs tamoxifen $360) as well as adverse effects caused by IL-2/IFN-alpha are also factors of importance. The study emphasizes the need for more effort to find the 'optimal schedule' of immunotherapy, as well as the need for randomized controlled studies before approval of a new treatment in the routine setting.


British Journal of Cancer (1998) 77(8), 1311–1317
© 1998 Cancer Research Campaign

### Survival in renal cell carcinoma – a randomized evaluation of tamoxifen vs interleukin 2, α-interferon (leucocyte) and tamoxifen

**R Henriksson[1], S Nilsson[2], S Colleen[3], P Wersäll[4], M Helsing[5], R Zimmerman[6] and K Engman[7]**

[1]Department of Oncology, Umeå University Hospital; [2]Department of Oncology, Uppsala University Hospital; [4]Karolinska Institute, [6]Södersjukhuset, Stockholm, [5]Örebro Hospital; [3]Department of Urology, Lund University Hospital, Sweden; and [7]BioNative AB, Umeå, Sweden

**Summary** Metastatic renal cell carcinoma (RCC) has a poor prognosis. Conventional treatment strategies, including chemotherapy and hormonal therapy, have limited value. Although encouraging results have been achieved in terms of objective response using immunological manipulations, no conclusive studies yet exist with a controlled comparative evaluation of survival. Therefore, the present study was undertaken, which compared one of the present (and presumed best) treatments, interleukin 2/interferon-α (IL-2/IFN-α) and tamoxifen, with a control arm of tamoxifen only. Tamoxifen has been shown to potentiate in vivo anti-tumour activity of IL-2, and because of its non-toxic behaviour it was included in both groups. The study was open, randomized and included seven institutions in Sweden. The patients were stratified according to the different centres involved. An interim analysis was planned when a minimum of 100 patients were evaluable. The 128 patients finally included had a histologically documented metastatic RCC, with a life expectancy of more than 3 months, a performance status WHO 0–2 and no prior chemo- or immunotherapy. Informed consent was obtained from each patient. The patients randomized to the control arm ($n$ = 63) received only tamoxifen 40 mg p.o. daily for at least 1 year or until progression. The patients ($n$ = 65) randomized to biotherapy received subcutaneous recombinant IL-2, leucocyte IFN-α in a treatment cycle of 42 days, as well as tamoxifen p.o. In the absence of undue toxicity or disease progression, these patients received one additional treatment cycle of 42 days followed by maintenance treatment, consisting of 5 days therapy every 4 weeks, for 1 year, or until proven progression. Only two patients in the tamoxifen-only group received immunotherapy when the disease progressed, but without any beneficial effect. All patients received appropriate local treatment when indicated. The interim analysis demonstrated no survival advantage for either group, and therefore further inclusion of patients was stopped. The median follow-up was 11 months (range 0.4–48 months). The final survival analysis showed no significant differences between the two treatment arms in so far as comparison from the day of diagnosis of primary disease, from the day of first evidence of metastatic spread, or from the onset of treatment. This was valid both when the evaluation was performed with regard to intention to treat and when the analysis was directed only to patients that managed at least one treatment cycle (42 days) of IL-2/IFN-α. The adverse effects were more pronounced in the IL-2/IFN-α group. Although the number of patients is limited, the results raise doubt concerning immunotherapy with IL-2 and IFN-α as a routine treatment in the management of advanced RCC. The difference in cost of drugs and health care (drug costs per patient: IL-2/IFN-α $27000 vs tamoxifen $360) as well as adverse effects caused by IL-2/IFN-α are also factors of importance. The study emphasizes the need for more effort to find the 'optimal schedule' of immunotherapy, as well as the need for randomized controlled studies before approval of a new treatment in the routine setting.

**Keywords**: renal cell carcinoma; survival; tamoxifen; interleukin 2; α-interferon

Advanced renal cell carcinoma (RCC) is considered to be beyond curative treatment. Chemotherapy and radiotherapy are only used in the palliative setting, and chemotherapy alone is of limited value (Oliver, 1994; Savage, 1995; Wagstaff et al, 1995). Hormonal manipulation, such as medroxyprogesteron acetate and tamoxifen have, in some observations, caused objective responses of short duration (Harris, 1983; Linehan et al, 1989). RCC is one of the few tumours with a known propensity for spontaneous regression (Katz et al, 1982; Oliver et al, 1989). Host factors such as the immune system may, in certain circumstances, influence the outcome. A number of studies strongly suggest that interferon α (IFN-α) and interleukin 2 (IL-2), especially in combination, could be of therapeutic value (Rosenberg et al, 1993; Oliver, 1994; Atzpodien et al, 1995; Facendola, 1995; Savage, 1995; Wagstaff et al, 1995; Hänninen et al, 1996). Although these treatment options can induce objective responses, they are associated with significant side-effects and considerable expense (Oliver, 1994; Facendola et al, 1995; Savage, 1995; Wagstaff et al, 1995). At present, no conclusive studies exist that, in prospective controlled fashion, have evaluated the effects of immunological manipulations on survival in patients with RCC (Ljungberg and Henriksson, 1997). Therefore, this randomized, controlled study compared subcutaneously administered leucocyte IFN-α and IL-2 with peroral tamoxifen, with special emphasis on survival. Tamoxifen has been shown to cause objective responses and to potentiate in vivo activity of IL-2 (Kim et al, 1990; Stahl et al, 1991). Owing to its well-tolerated behaviour, it was included in both treatment arms.

*Received 23 April 1997*
*Revised 11 September 1997*
*Accepted 30 September 1997*

*Correspondence to:* R Henriksson

**Table 1** Inclusion and exclusion criteria for participation in the study

Inclusion criteria
1. Histologically documented evidence of advanced RCC.
2. No prior chemotherapy, immunotherapy or extensive radiotherapy in the last 4 weeks (6 weeks for nitrosoureas, mitomycin C)
3. Ambulatory performance status WHO scale ≤ 2
4. Age 18–75 years, and life expectancy greater than 3 months
5. WBC ≥ 3500; platelets ≥ 100 000; haematocrit ≥ 30%
6. Serum bilirubin ≤ 1.25 × and creatinine ≤ 1.50 × upper limit of the institutional normal range

Exclusion criteria
1. Any of the above criteria are not met
2. A significant history or current evidence of a symptomatic cardiovascular disease (in questionable cases a stress test should be performed), haematopoietic, pulmonary, hepatic or renal dysfunctions
3. Patients with phaeochromocytoma or glaucoma
4. Patients positive for HIV, hepatitis B surface antigen ($HB_sAg$) and/or presenting with chronic hepatitis
5. Evidence of serious active infectious requiring antibiotic therapy
6. Contraindications to use of pressor agents
7. Patients with major organ allografts (IL-2 may increase T-cell mediated rejection, immunosuppressive agents are likely to reduce efficacy of IL-2) or autoimmune disease.
8. Patients with clinical evidence of CNS metastases
9. Patients who require or are likely to require corticosteroids for intercurrent disease during the active treatment with IL-2/IFN-α and/or tamoxifen
10. Patients with know seizure disorders or CNS disease
11. Pregnant or lactating women
12. Patients with second primary malignancies, concurrently
13. Patients who do not give their inform consent

### MATERIAL AND METHODS

### Study objectives

The primary objective of this study was to compare the effects of subcutaneously administered IL-2, IFN-α and tamoxifen with tamoxifen alone in terms of *overall survival* in patients with advanced RCC.

### Study design

The study was an open, randomized, multicentre study including seven major institutions in Sweden and was performed between 1992 and 1995. Inclusion and exclusion criteria are outlined in detail in Table 1.

Two hundred patients were originally planned to be included in the study. It was estimated that it would provide a power of 70% certainty to detect a difference at the 5% level in increasing mean survival from 10 months to 22 months. The patients were stratified according to the different centres involved. An interim analysis was intended to be performed when a minimum of 100 patients were evaluable. In the final analysis, a total of 128 patients were included in the study. In all, 65 patients were treated with a combination of IL-2, IFN-α and tamoxifen, and 63 patients with tamoxifen alone (for details, see Table 2). One patient in each group was older than stated in the inclusion criteria (77 and 78 years old respectively) and included by mistake. They were included in the analysis.

### Study drugs and treatment schedule

Patients were randomized to a combined treatment with recombinant IL-2 (Proleukin, EuroCetus, Division of Chiron, Emeryville CA, USA), natural leucocyte interferon-α (Interferon AlfaNative, BioNative AB, Umeå, Sweden) (Ahrén et al, 1992) and tamoxifen (Tamaxin, Orion-Pharma, Sweden) *or* to tamoxifen alone. All patients were treated with tamoxifen 40 mg p.o. daily. Patients allocated to the combination therapy were also treated with regularly repeated treatment cycles, including both IL-2 and IFN-α (Table 3) according to a schedule reported previously by Atzpodien and colleagues (1990). The treatment was given on an outpatient basis and the patients usually managed their own injections. Concomitant treatment with corticosteroids was not allowed.

Forty of the 65 patients in the immunotherapy group were estimated to have received 75% or more of the planned total dose of IL-2 and IFN-α. Five patients received less than 25% of the planned total dose.

### Clinical assessments and laboratory examinations

After all inclusion and exclusion criteria were fulfilled, a general physical examination and routine test for determination of haematological and biochemical parameters were performed before enrolment, and then repeated during every new treatment cycle throughout the study. When clinical evidence of disease progression was obtained, a thorough radiological examination was performed. In the case of a clear clinical and radiological deterioration, the treatment was interrupted.

### Adverse reactions

Toxicity was graded according to the WHO criteria, i.e. it was graded as mild (grade 1), moderate (grade 2), severe (grade 3) and life threatening (grade 4). IL-2 and IFN-α were reduced by 50% in patients when grade 3 or 4 toxicity was present (Table 4).

### Relapse treatment

The treatment options in the relapse situation was left to the discretion of each responsible physician, but it was recommended to use modalities other than immunotherapy. As only two patients in the tamoxifen only group received immunotherapy (IFN-α) because of

© Cancer Research Campaign 1998

**Table 2** Patients' characteristics at the time of inclusion in the study

| | Tamoxifen | IL-2/IFN-α/tamoxifen |
|---|---|---|
| Total no of patients enrolled | 63 | 65 |
| Sex (male/female) | 49/14 | 43/22 |
| Age median (range) | 63 (32–78) | 61 (43–77) |
| Previous nephrectomy | 52 | 54 |
| Number of patients with metastasis | | |
| Multiple location | 39 | 37 |
| 1–3 metastasis | 21 | 20 |
| Lung >5 metastasis | 23 | 19 |
| Lung | 45 | 32 |
| Liver | 6 | 11 |
| Bone | 18 | 15 |
| Lymphoid glands | 19 | 19 |
| Local relapse | 5 | 8 |
| Laboratory values (mean s.e.m.) | | |
| Hb | 128 (2.7) | 127 (2.6) |
| Platelets | 295 (18) | 342 (20) |
| WBC | 11 (4.3) | 8.9 (1.2) |
| Creatinin | 111 (3.8) | 103 (3.4) |
| Albumin | 39 (0.8) | 38 (0.7) |

**Table 3** Schedule of recombinant IL-2 (Proleukin) + IFN-α (Interferon Alfanative)

A complete course of treatment on the used protocol comprised 6 weeks of s.c. IL-2 followed by 2–3 weeks' rest. Patients received s.c. IL-2 $4.8 \times 10^6$ IU $m^{-2}$ per single dose given q 8 h on days 1 and 22, and q 12 h on day 2 and 23, followed by $2.4 \times 10^6$ IU $m^{-2}$ q 12 h on day 3–5, 8–12, 15–19, 24–26, 29–33 and 36–40

In addition, patients received IFN-α at $3 \times 10^6$ IU $m^{-2}$ on days 3, 5, 24, 26 and at $6 \times 10^6$ IU $m^{-2}$ on days 8, 10, 12, 15, 17, 19, 29, 31, 33, 36, 38 and 40

Maintenance treatment
Five days' therapy every 4 weeks up to 1 year or until disease progression or undue toxicity
Daily: IL-2 at $2.4 \times 10^6$ IU $m^{-2}$ q 12 h
Day 1, 3 and 5: IFN-α $3 \times 10^6$ IU $m^{-2}$

**Table 4** Survival values – intention to treat

| | Tamoxifen | IL-2/IFN-α/tamoxifen |
|---|---|---|
| From randomization | 13.3 (0.4–25.7) (8.4–18.2) | 11.8 (0.5–28.9) (8.5–15.2) |
| From diagnosis | 27.8 (1.4–180.3) (10.8–44.8) | 30.2 (2.1–195.0) (14.7–45.7) |
| From metastasis | 22.6 (1.4–114.0) (13.5–31.6) | 18.0 (1.2–154.5) (8.6–27.4) |

Median, range and 95% confidence interval – no significant differences. Expressed in months.

progressive disease and both patients displayed continuous progressive disease, these patients were included in the final analysis of intention to treat. Three patients in the IL-2/IFN-α arm received a further course of IFN-α and IL-2. Radiotherapy was given mainly as pain palliation in 16 patients in the tamoxifen group and in 14 patients in the IL-2/IFN-α tamoxifen group. One patient in each group received chemotherapy without any obvious effect.

### Ethics

The study conformed to the Helsinki/Tokyo Declaration, and was approved by the Ethics Committees at the University Hospital, Umeå, Uppsala, Lund, Örebro and Karolinska Institute, Stockholm. All patients were given verbal and written information about the study, and informed consent was obtained from each patient.

### Statistics

Statistical evaluation was performed in blinded fashion by an independent statistician. To estimate the probability of survival, the Kaplan–Meier method was used. Differences in survival between the two groups were tested using the log-rank test.

### RESULTS

### Interim analysis

An interim analysis was initiated when 100 patients were evaluated. The analysis was performed by two independent investigators (not involved in the study), who were directed to evaluate any differences in the survival between the two treatment arms. As there were no signs of survival advantage, and it was not probable

© Cancer Research Campaign 1998

that a further inclusion of patients could display a clinically meaningful difference, it was recommended that the study should be interrupted. The differences in adverse reactions between the two treatment arms were also of importance in this decision.

### Final analysis

When it was decided to stop the recruitment of patients, 128 patients had been randomized and included. The median follow-up time was 11 months from randomization (range 0.4–48 months). Within the first month after inclusion in the study, two patients had died in each of the treatment groups.

The survival (mortality) curves displayed no signs of statistically significant differences between the two treatment schedules (intention to treat), regardless of whether the analysis was performed from the day of randomization, from the day of primary diagnosis, or from the day of first evidence of metastasis (Figure 1). Nor were there any differences if only patients included in the survival analysis had received at least one treatment cycle of IL-2 and IFN-α (Figure 1 A–C inlets). There were no significant differences in mean or median survival of the patients in the two arms (Table 4). Twenty-six patients in the IL-2/IFN arm and 30 patients in the tamoxifen-only treated group were alive 1 year after initiation of the treatment. There were no significant differences in long-term survival. However, the number of patients in this long follow-up was limited (Figure 1). It must be emphasized that the response evaluation was not of primary concern; however, we observed complete and partial responders in both groups. Five complete responders (CRs) were seen in the IL-2/IFN-α group. Two CRs were obviously seen in the tamoxifen only group. Of these patients, the time to verified progression was 20–48+ months in the tamoxifen only group and 30–54+ months in the immunotherapy group.

The toxicities observed in this trial were similar to those expected from other trials. Virtually all patients in the IL-2/IFN-α tamoxifen group experienced some degree of a flu-like syndrome, including malaise and fever. Overall, the toxicity was graded 0–2 according to the WHO toxicity scale in the majority of the patients. However, grade 3–4 toxicity was seen in a substantial amount of patients and was more common in the IL-2/IFN-α-treated patients (Table 5).

### DISCUSSION

Metastatic RCC has a poor prognosis with a 5-year survival of less than 10%. With only slight or no efficacy with conventional chemotherapy and hormonal therapy, immunological manipulations have attracted major interest (Rosenberg et al, 1993; Besana et al, 1994; Oliver, 1994; Atzpodien et al, 1995; Facendola et al, 1995; Savage, 1995; Wagstaff et al, 1995; Hänninen et al, 1996). Recombinant cytokines, notably IL-2 and IFN-α, appear to have had encouraging results, and have led to approval by several national drug agencies in the management of RCC. However, in these studies, only objective responses were evaluated and the results compared with historic control patients (Savage, 1995). Therefore, it is of interest that the present study, which was focused on survival analysis, could not display any detectable differences in overall survival between a combination of IL-2, IFN-α and tamoxifen with only tamoxifen. The results are especially noteworthy when considering the adverse effects of the treatment and the higher expense of the combined treatment arm.

The results from the present study could be questioned because of the relatively limited number of patients included in the final analysis. Nevertheless, as outlined and interpreted by the investigators responsible for the interim analysis, a statistically significant survival difference between the treatments could not be detected unless considerably more patients were included. A drawback of many of the previously reported trials is the conclusion that the response rate was superior to 'historic controls', even although these trials were not randomized, and often did not use case-matched controls. Such drawbacks were emphasized by Philip and colleagues (1993) in a single-institution analysis of patients referred to their institution, thus further proving the importance for randomized trials with concurrent controls. It is also well known that patients with skeletal and/or liver metastases, as a group, respond less favourably than patients with, for example, only lung and/or lymph node metastases. Therefore, another alternative could have been to include only this latter patient category in this study. This protocol, however, investigated the general applicability of the therapy concept in the management of performance status patients (WHO 0–2) with metastasized RCC. This approach was facilitated by the recruitment of consecutive patients and the randomized design of the study. In addition to the observation that survival was similar in the two patient groups, even in respect to long-term survival, it is of importance to emphasize that the survival was quite comparable with survival data reported in previous studies of RCC patients treated with IL-2/IFN. In fact, median survival in each of the treatment groups (see Table 4) is better than that seen in the Swedish registry studies of advanced RCC patients. Moreover, the median survival has also been shown in other IL-2/IFN studies to be at the same level at 12 months (Facendola et al, 1995).

Factors that could affect the outcome for patients with metastatic disease include time from initial diagnosis, weight loss and the number of metastatic sites involved (Elson et al, 1988). However, this evaluation of survival did not seem to be different, regardless of the time frame of the analysis (from the date of primary diagnosis or the start of the treatment or from the time of first signs of metastatic spread). There was no obvious initial variation in laboratory parameters or metastatic spread of the disease (see Table 2), which further reduces the risk of differences in prognostic factors between the treatment groups. The use of natural interferon-α decreases the likelihood of neutralizing antibodies (Prümmer, 1993), and no antibodies against IFN-α in repeated and continuous analysis throughout the study were detected.

Previously published studies have also suggested that immunotherapy has no impact on survival (Steineck et al, 1990; Wagstaff et al, 1995). In this context it is also of interest to emphasize that controlled randomized trials evaluating different approaches of immunotherapy on survival, for example IL-2 vs IL-2 plus lymphokine-activated killer cells or IFN-α vs IFN-α and vinblastin, displayed no significant differences (Fosså et al, 1992; Rosenberg et al, 1993; Kellokumpu-Lehtinen et al, 1995). Furthermore, the advantage of an increased response rate after immunological manipulation has not always been translated to a better chance of survival (Fosså et al, 1992; Facendola et al, 1995). This could imply that all the survival curves and the supposed effects of immunotherapy simply highlighted the natural history of the disease. This was further highlighted by the fact that no differences could be seen in survival between IFN-γ1b and placebo in a randomized study including 197 patients (Elhilai et al, 1997).

© Cancer Research Campaign 1998

**Figure 1** Kaplan–Meier survival. Time to death from (**A**) randomization, (**B**) day of primary diagnosis and (**C**) day of first evidence of metastatic spread. Large figures denote intention to treat and inlets include in the IL-2/IFN-α schedule only those patients that have received at least 42 days (one cycle) treatment. —, IL-2/IFN-α; – – –, tamoxifen-only treatment

**Table 5** Number of patients who suffered from WHO grade 3–4 toxicity during treatment

| | Tamoxifen | IL-2/IFN-α/tamoxifen |
|---|---|---|
| Total number of patients enrolled | 63 | 65 |
| Fatigue | 19 | 38 |
| Anorexia | 7 | 14 |
| Nausea | 5 | 14 |
| Fever | 0 | 8 |
| Diarrhoea | 2 | 5 |
| Myalgia | 14 | 12 |
| Pulmonary | 11 | 9 |
| Infection | 0 | 2 |
| Cutaneous | 0 | 1 |
| Headache | 0 | 2 |
| Oral | 1 | 1 |
| CNS | 2 | 1 |

Spontaneous remissions were also seen in the placebo arm, thus underlining the variable natural history of metastatic RCC. Substantial improvement was recently shown in response rate over other regimens in patients with progressing RCC (Hänninen et al, 1996) who, in a non-randomized evaluation, underwent alternating cycles of combination of immunotherapy (IL-2 and IFN-α) and chemoimmunotherapy (5-fluorouracil and IL-2). Once again, in this study the overall response rate (CR+PR) of 39% was not directly related to long-lasting remissions. The beneficial effect of chemoimmunotherapy (IL-2, IFN-α and 5-FU) was further proposed in a small randomized study against tamoxifen (Atzpodien et al, 1997). It is also of interest to recall that a survival benefit was seen for patients treated with cimetidine plus autolymphocyte therapy compared with cimetidine only (Osband et al, 1990). Similar results have been seen when RCC patients with nodal disease were given adjuvant therapy (Sawczuk et al, 1997).

Immunological manipulation, even with the present subcutaneous regimen, is associated with toxicity, at least during the treatment period. The toxicity seen was in accordance with earlier reports. The present study also included a substantial number of patients with grade 3 and 4 toxicity in the IL-2- and IFN-α-treated patients. Thus, when discussing any beneficial effects, these aspects must also be considered. In evaluation of toxicity of a given treatment, it must also be considered that a local tumour progression could explain a deterioration. The observed CNS and lung toxicity in the tamoxifen group most probably were due to local tumour growth.

Hormone manipulation has been used for the treatment of RCC for many years. The initial high response rate of medroxyprogesterone acetate has, with modern response criteria, been modified and is now established at approximately 2% (Harris, 1983; Linehan et al, 1989), and with no proven effect on survival. In the present study, objective responses were observed including two complete responses with tamoxifen-only treatment, and several long-lasting remissions/stable diseases were also seen. Note that these patients never received immuno- or chemotherapy. Previously, tamoxifen in higher doses than used in this study has also been shown to cause objective responses and even complete responses in advanced RCC (Stahl et al, 1991). Nevertheless, even if it cannot be established that tamoxifen has any clear-cut effects on survival, tamoxifen because of its non-toxic features can be used in the palliative setting. The role of endocrine manipulation might at least be re-evaluated, especially in the context that hormone treatment can interact with the immune system (Oliver, 1994). As a large number of objective responses and some long-term survivors have been seen, even in this study over the years, there might be a subpopulation of patients that could benefit from immunotherapy (or hormone manipulation). Future studies are therefore of interest to find methods to identify those patients.

In summary, even if the number of patients is relatively limited, the present study questions the benefit of immunotherapy with IL-2 and IFN-α as a *routine* treatment in the management of advanced RCC. The study suggests the use of a less toxic regimen, for example tamoxifen, in the routine setting and as an adequate control in further trials. This assumption is also strengthened by the difference in costs between the two treatment arms (drug costs only: IL-2/IFN-α $27 000 vs $360 per patient in the tamoxifen-only group). However, the chemoimmunotherapy approach with and without 13-*cis*-retinoic acid (Motzer et al, 1995; Hänninen et al, 1996; Atzpodien et al, 1997) is exciting and suggests that unexpected regimens might exist that are better than any thus far tested. It is obvious that there is much need for investigation to find 'the optimal' application for immunotherapy in the clinical setting.

### ACKNOWLEDGEMENTS

The participating investigators were: Per Bergström, Department of Oncology, Umeå University Hospital, Umeå, Sweden; Christina Wedelin, Radiumhemmet, Karolinska sjukhuset, Stockholm; Izabela Nilsson, Department of Oncology, Malmö Hospital, Malmö. Statistical evaluation was by Björn Tavelin, Oncology Centre, Umeå University, S-901 85 Umeå, Sweden. The Interim analysing committee was Professor Hans Strander and Associate Professor Giuseppe Massucci, Department of Oncology, Karolinska Institute, S-171 76 Stockholm, Sweden. The study was sponsored by BioNative AB and Orion-Pharma, Sweden and was monitored by Katarina Engman, BioNative AB, Umeå, Sweden.

### REFERENCES

Ahrén B, Engman K and Lindblom A (1992) Treatment of malignant midgut carcinoid with a highly purified human leukocyte α-interferon. *Anticancer Res* **12**: 129–134

Atzpodien J, Körfer A, Franks CR, Poliwoda H and Kirchner H (1990) Home therapy with recombinant interleukin-2 and interferon-α2b in advanced human malignancies. *Lancet* **335**: 1509–1512

Atzpodien J, Hänninen LE, Kirchner H, Bodenstein H, Pfreundschuh M, Rebmann U, Metzner B, Illiger H-J, Jakse G, Niesel T, Scholz H-J, Wilhelm S, Pielmeier T, Zabrewski G, Blum G, Beier J, Müller G-W, Duensing S, Anton P, Allhoff E, Jonas U and Poliwoda H (1995) Multiinstitutional home-therapy trial of recombinant human interleukin-2 and interferon alfa-2 in progressive metastatic renal cell carcinoma. *J Clin Oncol* **13**: 497–501

Atzpodien J, Kirchner H, Franzke A, Wandert T, Probst M, Buer J, Duensing S and Ganser A (1997) Results of a randomized clinical trial comparing SC interleukin-2, SC alpha-2a-interferon, and IV bolus 5-fluorouracil against oral tamoxifen in progressive metastatic renal cell carcinoma patients (abstract). *Proc ASCO* **16**: 1164

Besana C, Borri A, Bucci E, Citterio G, Di Lucca G, Fortis C, Matteucci P, Tognella S, Tresoldi M, Baiocchi C, Landonio G, Ghislandi E and Rugarli C (1994) Treatment of advanced renal cell cancer with sequential intravenous recombinant interleukin-2 and subcutaneous alpha-interferon. *Eur J Cancer* **30A**: 1292–1298

Elhilai M, Gleave M, Fradet Y, Davis I, Venner P, Saad F, Klotz L, Sanders C, Bajamonde A and Paton V (1997) Placebo-associated remissions in a multicenter randomized, double blind trial of interferon gamma 1b (rIFN-) for the treatment of metastatic renal cell carcinoma (mRCC) (abstract). *Proc ASCO* **16**: 1187

Elson PJ, Witte RS and Trump DL (1988) Prognostic factors for survival in patients with recurrent or metastatic renal cell carcinoma. *Cancer Res* **48**: 7310–7313

© Cancer Research Campaign 1998

Facendola G, Locatelli MC, Pizzocaro G, Piva L, Pegoraro C, Bobbio Pallavicini E, Signaroldi A, Meregalli M, Lombaardi F, Beretta GD, Scanzi F, Labianca R and Luporini G (1995) Subcutaneous administration of interleukin 2 and interferon-alpha-2b in advanced renal cell carcinoma: a confirmatory study. *Br J Cancer* **72**: 1531–1535

Fosså SD, Martinelli G, Otto U, Schneider G, Wander H, Oberling F, Bauer HW, Achtnicht U and Holdener EE (1992) Recombinant interferon alfa-2a with or without vinblastine in metastatic renal cell carcinoma: Results of a European multi-center phase III study. *Ann Oncol* **3**: 301–305

Hänninen LE, Kirchner H and Atzpodien J (1996) Interleukin-2 based home therapy of metastatic renal cell carcinoma: Risks and benefits in 215 consecutive single institution patients. *J Urol* **155**: 19–25

Harris DT (1983) Hormonal therapy and chemotherapy of renal carcinoma. *Semin Oncol* **10**: 422–430

Katz SE and Schapira HE (1982) Spontaneous regression of genitourinary cancer – an update. *J Urol* **128**: 1–4

Kellokumpu-Lehtinen P, Jantunen I, Flander M, Johansson R, Johansson R and Nordman E (1995) Combined interferon and vinblastine treatment in advanced renal cell cancer. *Acta Oncol* **34**: 975–977

Kim B, Warnaka P and Konrad C (1990) Tamoxifen potentiates in vivo antitumor activity of interleukin-2. *Surgery* **108**: 139–145

Linehan WM, Shipley WJ and Parkinson DR (1989) Cancer of the kidney and ureter. In: *Principles and Practice of Oncology*, De Vita VT, Hellman S and Rosenberg SA (eds), pp. 279–1005. J B Lippincott: Philadelphia

Ljungberg B and Henriksson R (1997) Immunotherapy of renal cell carcinoma. *Curr Opinion Urol* **7**: 252–258

Motzer RJ, Schwartz L, Murray Law T, Murphy BA, Hoffman AD, Albino AP, Vlamis V and Nanus DM (1995) Interferon alfa-2a and 13-cis-retinoic acid in renal cell carcinoma: Antitumor activity in a phase II trial and interactions in vitro. *J Clin Oncol* **13**: 1950–1957

Oliver RTD (1994) Renal cell cancer: is there long-term survival advantage from cytokine treatment? *Eur J Cancer* **30A**: 1214–1216

Oliver RTD, Nethersell ABW and Bottomley JM (1989) Unexplained spontaneous regression and alpha-interferon as treatment for metastatic renal carcinoma. *Br J Urol* **63**: 128–131

Osband ME, Lavin PT, Babayan RK, Graham S, Lamm DL, Parker B, Sawczuk I, Ross S and Krane RJ (1990) Effect of autolymphocyte therapy on survival and quality of life in patients with metastatic renal-cell carcinoma. *Lancet* **335**: 994–998

Philip T, Negrier S, Lasset C, Coronel B, Bret M, Baly JY, Merrouche Y, Carrie C, Kaemmerlein P, Chauvin F, Favrot M, Oskam R, Tabah I, Clavel M, Moskovtchenko JF and Mercatello A (1993) Patients with metastataic renal carcinoma candidate for immunotherapy with cytokines. Analysis of a single institution study on 181 patients. *Br J Cancer* **68**: 1036–1042

Prümmer O (1993) Interferon-alpha antibodies in patients with renal cell carcinoma treated with recombinant interferon-alpha-2A in an adjuvant multicenter trial. *Cancer* **71**: 1828–1834

Rosenberg SA, Lotze MT, Yang JC, Topalian SL, Chang AE, Schwartzentruber DJ, Aebersold P, Leitman S, Linehan WM, Seipp CA, White DE and Steinberg SM (1993) Prospective randomized trial of high-dose interleukin-2 alone or in conjunction with lymphokine-activated killer cells for the treatment of patients with advanced cancer. *J Natl Cancer Inst* **85**: 622–632

Savage PD (1995) Renal cell carcinoma. *Curr Opin Oncol* **7**: 275–280

Sawczuk IS, Graham SD Jr, Miesowicz F, the ALT Adjuvant Study Group (1997) Randomized controlled trial of adjuvant therapy with ex vivo activated T cells (ALT) in $T_{1-2a,b,c}$ or $T_2N_{+1}M_0$ renal cell carcinoma (abstract). *Proc ASCO* **16**: 1163

Stahl M, Schmoll E, Becker H, Schlichter A, Hoffman L, Wagner H, Possinger K, Müller W, Köllermann M, Weidenhammer W and Schmoll H-J (1991) Lopidamine versus high-dose tamoxifen in progressive, advanced renal cell carcinoma: results of an ongoing randomized phase II study. *Semin Oncol* **18**: 33–37

Steineck G, Strander H, Carbin B-E, Borgström E, Wallin L, Achtnich U, Arvidsson A, Söderlund V, Näslund I, Esposti P-L and Norell SE (1990) Recombinant leukocyte interferon alpha-2a and medroxyprogesterone in advanced renal cell carcinoma. A randomized trial. *Acta Oncol* **29**: 155–162

Wagstaff J, Baars JW, Wolbink G-J, Hoekman K, Eerenberg-Belmer AJM and Hack CE (1995) Renal cell carcinoma and interleukin-2: A review. *Eur J Cancer* **31A**: 401–408

© Cancer Research Campaign 1998